# Linking a European cohort of children born with congenital anomalies to vital statistics and mortality records: A EUROlinkCAT study

**DOI:** 10.1371/journal.pone.0256535

**Published:** 2021-08-27

**Authors:** M. Loane, J. E. Given, J. Tan, A. Reid, D. Akhmedzhanova, G. Astolfi, I. Barišić, N. Bertille, L. B. Bonet, C. C. Carbonell, O. Mokoroa Carollo, A. Coi, J. Densem, E. Draper, E. Garne, M. Gatt, S. V. Glinianaia, A. Heino, E. Den Hond, S. Jordan, B. Khoshnood, S. Kiuru-Kuhlefelt, K. Klungsøyr, N. Lelong, L. R. Lutke, A. J. Neville, L. Ostapchuk, A. Puccini, A. Rissmann, M. Santoro, I. Scanlon, G. Thys, D. Tucker, S. K. Urhoj, H. E. K. de Walle, D. Wellesley, O. Zurriaga, J. K. Morris

**Affiliations:** 1 Faculty of Life and Health Sciences, Ulster University, Northern Ireland, United Kingdom; 2 Population Health Research Institute, St George’s, University of London, London, United Kingdom; 3 OMNI-Net for Children International Charitable Fund, Rivne Regional Medical Diagnostic Center, Rivne, Ukraine; 4 Emilia Romagna Registry of Birth Defects, University Hospital of Ferrara, Ferrara, Italy; 5 Klinika za dječje bolesti, Zagreb, Croatia; 6 Institut National de la Santé et de la Recherche Médicale, Paris, France; 7 Rare Diseases Research Unit, Foundation for the Promotion of Health and Biomedical Research in the Valencian Region, Valencia, Spain; 8 Departamento de Salud Gobierno Vasco, Basque Country, Spain; 9 Unit of Epidemiology of Rare Diseases and Congenital Anomalies, Institute of Clinical Physiology, National Research Council, Pisa, Italy; 10 Biomedical Computing Limited, Battle, United Kingdom; 11 East Midlands & South Yorkshire Congenital Anomaly Registry, University of Leicester, Leicester, United Kingdom; 12 Hospital Lillebaelt, Region Syddanmark, Denmark; 13 Directorate for Health Information and Research, G’Mangia, Malta; 14 Faculty of Medical Sciences, Population Health Sciences Institute, Newcastle University, Newcastle upon Tyne, United Kingdom; 15 Finnish Institute for Health and Welfare, Helsinki, Finland; 16 Provinciaal Instituut voor Hygiëne (PIH), Antwerpen, Belgium; 17 Swansea University, Wales, United Kingdom; 18 Division of Mental and Physical Health, Department of Global Public Health and Primary Care, Norwegian Institute of Public Health, University of Bergen, Bergen, Norway; 19 Department of Genetics, University Medical Center, University of Groningen, Groningen, The Netherlands; 20 Territorial Care Service, Emilia Romagna Health Authority, Bologna, Italy; 21 Medical Faculty Otto-von-Guericke, Malformation Monitoring Centre Saxony-Anhalt, University Magdeburg, Magdeburg, Germany; 22 Public Health Wales, Wales, United Kingdom; 23 Section of Epidemiology, University of Copenhagen, Copenhagen, Denmark; 24 Wessex Clinical Genetics Service, Princess Anne Hospital, Southampton, United Kingdom; Center of Pediatrics, GERMANY

## Abstract

EUROCAT is a European network of population-based congenital anomaly (CA) registries. Twenty-one registries agreed to participate in the EUROlinkCAT study to determine if reliable information on the survival of children born with a major CA between 1995 and 2014 can be obtained through linkage to national vital statistics or mortality records. Live birth children with a CA could be linked using personal identifiers to either their national vital statistics (including birth records, death records, hospital records) or to mortality records only, depending on the data available within each region. In total, 18 of 21 registries with data on 192,862 children born with congenital anomalies participated in the study. One registry was unable to get ethical approval to participate and linkage was not possible for two registries due to local reasons. Eleven registries linked to vital statistics and seven registries linked to mortality records only; one of the latter only had identification numbers for 78% of cases, hence it was excluded from further analysis. For registries linking to vital statistics: six linked over 95% of their cases for all years and five were unable to link at least 85% of all live born CA children in the earlier years of the study. No estimate of linkage success could be calculated for registries linking to mortality records. Irrespective of linkage method, deaths that occurred during the first week of life were over three times less likely to be linked compared to deaths occurring after the first week of life. Linkage to vital statistics can provide accurate estimates of survival of children with CAs in some European countries. Bias arises when linkage is not successful, as early neonatal deaths were less likely to be linked. Linkage to mortality records only cannot be recommended, as linkage quality, and hence bias, cannot be assessed.

## Introduction

Congenital anomalies are structural anomalies and genetic syndromes that occur during development of the embryo and are a leading cause of perinatal and infant mortality in Europe [1]. Around 2–3% of all children born in Europe every year will have a major congenital anomaly (CA). The European surveillance of congenital anomalies (EUROCAT) network of population-based CA registries provides essential epidemiologic information and surveillance on CAs in Europe but information is mainly collected up to a baby’s first year of life [2–4]. There is little information on survival after one-year of age in Europe [5], with studies either analysing all anomalies combined [6] or concentrating on a few specific anomalies, such as spina bifida or Down syndrome [7,8]. One study investigated 20-year survival for a range of CAs in the North of England, but was unable to report survival for many rare CAs due to small numbers [9].

Death certificates are a reliable source of information on the number of deaths, as all deaths must be registered. However, although the primary cause of death such as infection is listed, a US study found that CAs are often not listed as an underlying cause of death [10]. This means that death certificates may not be an accurate source of information on the causes of death in children with CAs. For example, the death certificate of a child with microcephalus who died as a result of an infection may list the infection as a cause of death, but the underlying condition i.e. microcephalus is not stated. Copeland et al. [10] concluded that the only way to accurately assess mortality and survival in children with rare anomalies is to pool data across CA registries and link these to death certificates. Using such methods, a study from the US for children born 1992–1998 found that mortality of children with CAs up to age 7 years was over seven times higher than the mortality in children without CAs [11]. Many countries in Europe have linked to death records to investigate perinatal mortality, but linking to death records as a method of assessing survival of older children across Europe has not been previously reported [12].

One aim of the EUROlinkCAT study is to investigate the survival of children with specific CAs for the first 10 years of their lives by linking livebirths with CAs in EUROCAT registries to mortality records from various administrative sources. This study reports on the quality and accuracy of linkage to national vital statistics or mortality records in order to provide information for future researchers considering conducting similar studies in other population groups.

## Materials and methods

### Design and setting

All CA registries who were members of EUROCAT (www.eurocat-network.eu) were invited to participate in the HORIZON 2020-funded EUROlinkCAT study. Initially, 20 registries from 12 countries agreed to try to link all livebirths with a CA in their region to mortality records up to their 10^th^ birthday (Table 1). An additional registry who had already linked their data also participated in EUROlinkCAT (Norway).

**Table 1:** Methods of linking by registry.

Country: Registry	Linkage to vital statistics or mortality	Source Data	Linkage Identifiers	Method
Belgium: Antwerp	Mortality records	Flemish Agency for Care and Health, Belgian Mortality records	Birth weight, infant sex, residence, birth date of mother (National ID numbers could not be used)	A third party conducted linkage of CA file to the Belgian Mortality records. Probabilistic linkage
Croatia: Zagreb	Mortality records	Republic of Croatia Bureau of Statistics	Unique identification number (OIB)	CAs using a unique identification number were sent to the National Statistics Bureau for information on mortality Manual linkage
Denmark: Funen	Vital statistics	Statistics Denmark (SD)	Pseudonymised personal ID (PNR)	SD created a pseudonymised personal ID (PNR) used to link information in different registers. A combination of deterministic and probabilistic linkage was used. The Child’s PNR did not link all the children and matching of maternal PNR, birth date, maternal age, gestational age, birth weight and sex were used to link these.
Finland	Vital statistics	Cause-of-Death Register held by Statistics Finland	Unique identification PIN number for each death registered	Registry conducted their own linkage between the Finnish Register of Congenital Anomalies and the Cause-of-Death Register held by Statistics Finland. Deterministic linkage
France: Paris	Vital statistics	Civil register and mortality records at the French National Institute of Statistics and Economic Studies (INSEE)	Unique ID	INSERM linked their CA dataset to the civil register and mortality records Deterministic linkage
Germany: Saxony-Anhalt	Mortality records	Death records	Birth month and year, infant sex, birth weight, birth year of mother, residence	Manually
Italy: Emilia Romagna	Vital statistics	Regional Mortality Registry (RMR), Regional Inhabitant Registry (RIR), and Report for National Institute of Statistics (ISTAT)	Unique identification number	CA cases were matched to the baby’s birth record data (CeDAP), the baby’s hospital record data (SDO) and the mother’s hospital record data (SDO) which was matched with the baby’s hospital data (SDO) which was then matched to the mortality record. Probabilistic linkage was used between the EUROCAT dataset and CeDAP. Deterministic linkage was used between CeDAP, SDO and Mortality datasets
Italy: Tuscany	Vital statistics	Regional Registry Office, Mortality database, Regional discharge database	Unique ID (unique identifier number) based on five variables (first name, last name, date of birth, place of birth, and sex)	Cases have a unique ID, which was used for linkage to all the regional health databases. Deterministic linkage
Malta	Mortality records	Malta Congenital Anomalies Register, Mortality Register	Unique identification number	Cases manually linked using unique identification number. Deterministic linkage
Netherlands: Northern Netherlands	Vital statistics	Central Bureau of Statistics (CBS, also known as Dutch Statistics)	Date of birth, sex, postal code, and year of validity of postal code used to obtain national identification number	The encrypted national identification number (rinnumber) is used to link all available datasets at CBS. Deterministic linkage
Norway	Vital statistics	Medical Birth Registry of Norway (MBRN), Cause of Death registry	Unique national ID number given at birth	Used a linked dataset that was originally created for another project. This dataset linked the Medical Birth Registry of Norway (MBRN) with the Cause of Death registry. Deterministic linkage
Spain: Basque Country	Mortality records	Registro de Mortalidad, Spanish mortality database	A case’s first name and its two surnames combined with different combinations of other variables (i.e. date of birth and sex of child)	A unique identifier that consists of key words (and phonetic translators) from a case’s first name and its two surnames combined with different combinations of other variables (i.e. date of birth and sex of child) was created so cases could be linked. Reviewed individually, manually if low confidence. Probabilistic linkage.
Spain: Valencian Region	Mortality records	Regional Mortality database, National Mortality database	Identification number, date of birth, name of child, and sex of child	The CA file was linked first with the Regional Mortality database and then with the National Mortality database (to capture deaths outside of the Valencian Region) Deterministic linkage
Ukraine	Mortality records	Mortality records at the State Statistics Service of Ukraine (Derzhkomstat), Newborn registry contained in the Regional Children Hospital Statistics	Child’s date of birth, child’s birth order in multiple births, mother’s date of birth, mother’s surname name, father’s surname, and child’s patronymics)	Registry linked their CA cases to the mortality records and the newborn registry. Deterministic linkage
UK: Thames Valley; East Midlands and South Yorkshire; Wessex	Vital statistics	Personal Demographics Service, Hospital Episode Statistics (HES) and HES-ONS linked mortality data	NHS Number, Child’s surname, given names, postcode, date of birth and gender	A demographic trace is performed on the supplied personal identifiers; traced individuals are passed to HES for extraction of civil registrations data. Both deterministic and probabilistic linkage methods are used
UK: Wales	Vital statistics	Secure Anonymised Information Linkage Databank (SAIL), Office for National Statistics (ONS), National Health System Wales Informatics Service (NWIS)	NHS Number, Child’s surname, forename, postcode, date of birth and gender	The SAIL databank linked datasets from ONS, Welsh Demographic Survey, and NWIS with the EUROCAT CA file, using an anonymised linking field which has been encrypted for its use within SAIL. Both deterministic and probabilistic linkage is used in the SAIL algorithm

CA = Congenital Anomaly; CeDAP = birth records; SDO = hospital data.

#### Population

All live births with a CA born between 1^st^ January 1995 and 31^st^ December 2014 in the areas surveyed by the CA registries were followed up to 10 years of age or to the study end date. Mortality records were obtained from 1^st^ January 1995 to 31^st^ December 2015 so that at least one-year survival could be estimated for the entire cohort of children with CAs.

#### Data available in the EUROCAT registries

In addition to personal identifiers, all EUROCAT registries collect a core set of data elements (see Guide 1.4 (https://eu-rd-platform.jrc.ec.europa.eu/eurocat/data-collection/guidelines-for-data-registration_en#inline-nav-2) which include diagnoses of CAs (see S1 File), date of birth, infant sex, maternal age, gestational age at delivery, birth weight, number of babies in the pregnancy and survival for the first week of life. Some registries also collect information on survival up to the first year of life and beyond. Other sociodemographic variables such as maternal education, marital status, and maternal country of birth were collected locally by some registries.

#### Data available for linkage

There were two different types of data available for linkage: (i) vital statistics containing civil registrations data such as birth and death registrations, where each liveborn baby would be expected to have a record; and (ii) mortality records containing only death registrations. Registries linking to vital statistics databases are able to determine the proportion of successful and unsuccessful matches; i.e. if a EUROCAT case is identified in vital statistics, a match has occurred; if a EUROCAT case is not identified in the vital statistics, a match has not occurred. However, when linking to mortality records the number of successful and unsuccessful matches cannot be quantified, as if a EUROCAT case is not identified in the mortality records, it is likely to be because the child is still alive, but it may also be because the linkage failed (a missed match).

### Methods of linkage

The method of linkage was generally electronic and determined by the institution providing the mortality data, who also specified the linkage identifiers (see Table 1). Some registries linked cases manually using an ID number. Independent of type of data source, there were two methods of electronic linkage: deterministic and probabilistic linkage. In deterministic linkage a match is said to occur when the values for a set of variables are identical in both data sets. Deterministic linkage is often based on just an identification number (ID) which uniquely identifies each individual in a country. Probabilistic linkage involves calculating the probability of agreement of several common identifying variables found in data files such as name, address and date of birth and a match is said to occur when the probability is over a fixed level (often 90%). Probabilistic methods are useful when data are incomplete (truncated names) or mistyped and are often employed after performing the deterministic method.

#### Assessment of quality of linkage

Linkage errors occur when an individual is matched to another person’s record (false match) or fails to be matched with their record (missed match). Researchers from Ulster University (UU) worked with registries to standardise their data to a common data model (CDM), details of which are given in an earlier paper (Protocol paper submitted). The use of a CDM enabled a central linkage quality syntax script to be developed by the St George’s, University of London (SGUL) team which were distributed to all registries to evaluate the accuracy of the linkage by comparing characteristics of matched and not matched records in order to identify any factors leading to missed matches. For example, deaths within the first day of life may be less likely to be linked if a unique ID was not allocated at birth. The institutions performing the linkage were asked to specify for each matched case if the match was considered “strong” (i.e. confidence in matching coded as excellent or good) or “weak” (i.e. confidence in matching coded as fair or poor), with guidance provided based on the combination of identifiers used. Some of the linking institutions used their own local definitions, usually based on a scoring system, as to what constituted a ‘strong’ or ‘weak’ match.

### Ethics

The EUROCAT registries have ethics permissions and procedures for routine surveillance, data collection and transmission of anonymised data to a central database, according to national guidelines. Local registries follow national legislation as to whether parental consent is needed for registration of babies with anomalies [13]. A common study protocol was provided to all EUROCAT registries, who were responsible for making any necessary local amendments and submitting to the relevant authorities for additional ethics and other permissions required to link their data and provide aggregate and analytic results to the Central Results Repository (CRR) at UU. This was a lengthy process in some countries as the original data collection did not include expectation or consent for the data to be used in research, and a new legal basis had to be established. UU obtained ethics permission for the CRR. Additional assurances and procedures were adopted by registries (for example, the publication of privacy notices) to ensure compliance with the General Data Protection Regulation (GDPR) which came into force on 25 April 2018 in EU countries. A checklist of minimum specifications for data storage/backup was completed by each registry.

### Statistical analysis

For registries that linked to vital statistics, the odds of linkage occurring were examined by fitting univariate logistic regression models to all EUROCAT cases being linked to vital statistics with linkage failure as the outcome and each of the specific factors measured in EUROCAT as the independent variable. For registries linking only to mortality records, the odds of known deaths in the EUROCAT data being identified in the mortality records were examined by fitting univariate logistic regression models to all known deaths amongst EUROCAT cases with linkage failure as the outcome and specific factors measured in EUROCAT as the independent variables.

The values for maternal age, gestational length, number of babies in the pregnancy, infant sex, and birth weight in the EUROCAT data were compared with those in the linked data. Maternal age was judged to agree if the values differed by 1 year or less, birth weight was judged to agree if the values differed by <100 g and gestational length was judged to agree if the values differed by less than 1 week.

#### Small number restrictions (statistical disclosure control)

Five countries had limitations on the release of aggregate data and analytic results if the numbers of births involved are very small. The Northern Netherlands released data if all exported results were rounded to the nearest five. Rounding all frequencies ensures that original numbers cannot be inferred. For Denmark, a few named researchers at SGUL and UU were allowed access to the aggregate data for the purpose of collating and including in pooled-analysis, on condition that it was securely stored and processed, that any individual results involving fewer than five people were not released; and that personal identification was not possible from any released results. The SAIL databank (Wales) provided data to the CRR with the requirement that aggregate data on fewer than five people were not released, and could not be calculated from any information in the public domain. The registry from Antwerp, Belgium could not release any information on three or fewer cases. NHS Digital (England) allows small numbers to be published if the analysis is national, otherwise numbers below eight need to be suppressed.

## Results

### Methods of linkage

Out of 21 registries who agreed to participate in the study and to link their data, one registry from Île de la Réunion was unable to obtain ethics permissions to perform the linkage. Five English registries received approval to link their data 3 years after the initial application to do so; at the time of writing only three registries have completed linkage and their results are reported in this paper. Table 1 gives details of the methods of linkage in the remaining 18 participating registries. Eleven registries linked to vital statistics sources and seven registries linked only to mortality records.

Seven registries linked using only deterministic methods. Six registries used a combination of deterministic and probabilistic methods i.e. they linked cases first using deterministic methods, and then resorted to probabilistic methods for unlinked cases. Two registries used probabilistic methods only. Three registries linked all cases manually to mortality records (Malta, Saxony Anhalt, and Zagreb). Zagreb could only obtain identifiers for 78% of cases, born between 2011 and 2014 hence the registry was excluded from survival analysis due to the potential for bias. Ukraine reviewed all their cases manually and Basque Country reviewed their cases in the first few years of data collection due to concerns about too few mortality records being linked.

### Success of linkage to vital statistics

**Fig 1:**
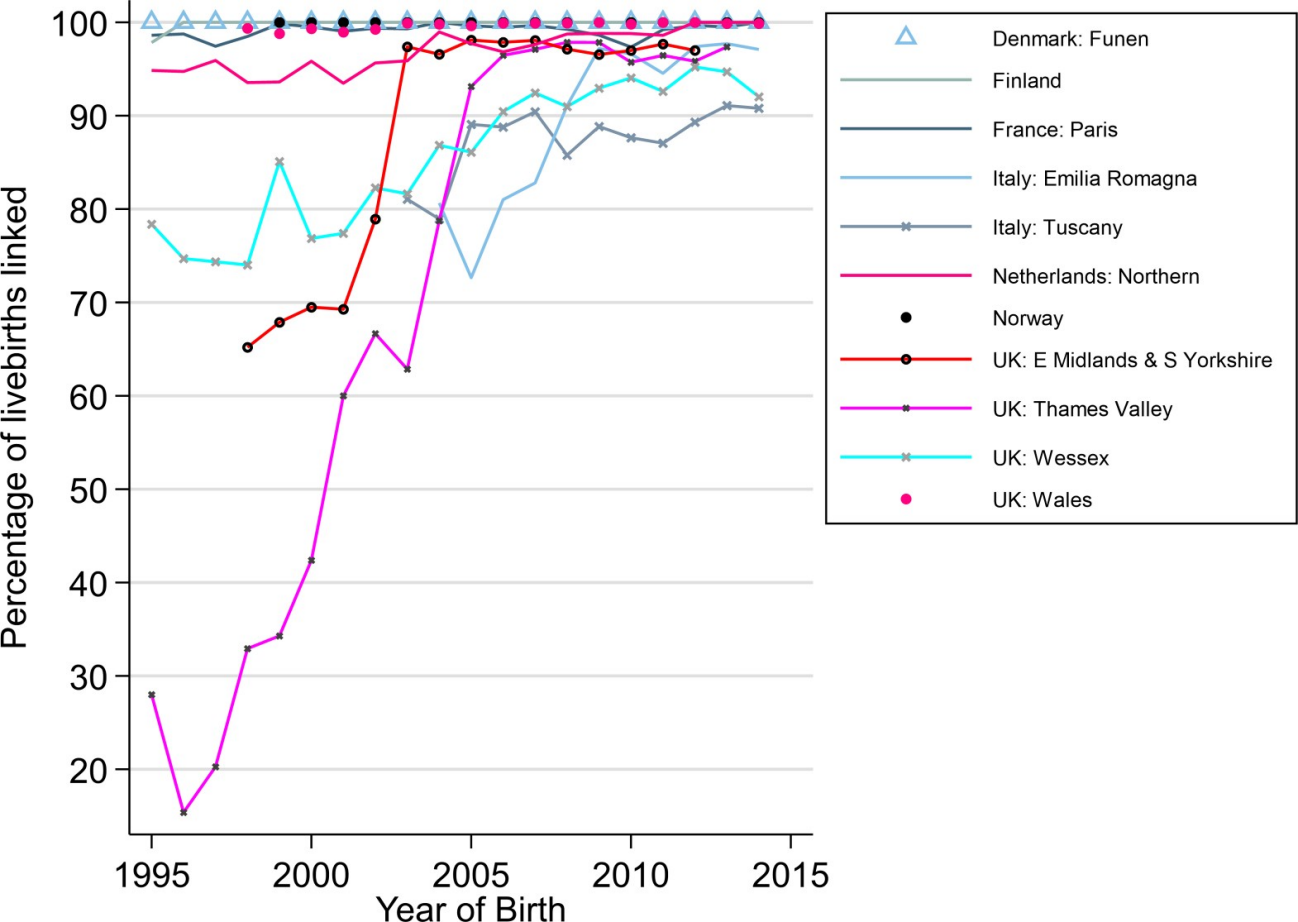
Percentage of live births linked to vital statistics in each registry by birth year.

**Fig 2:**
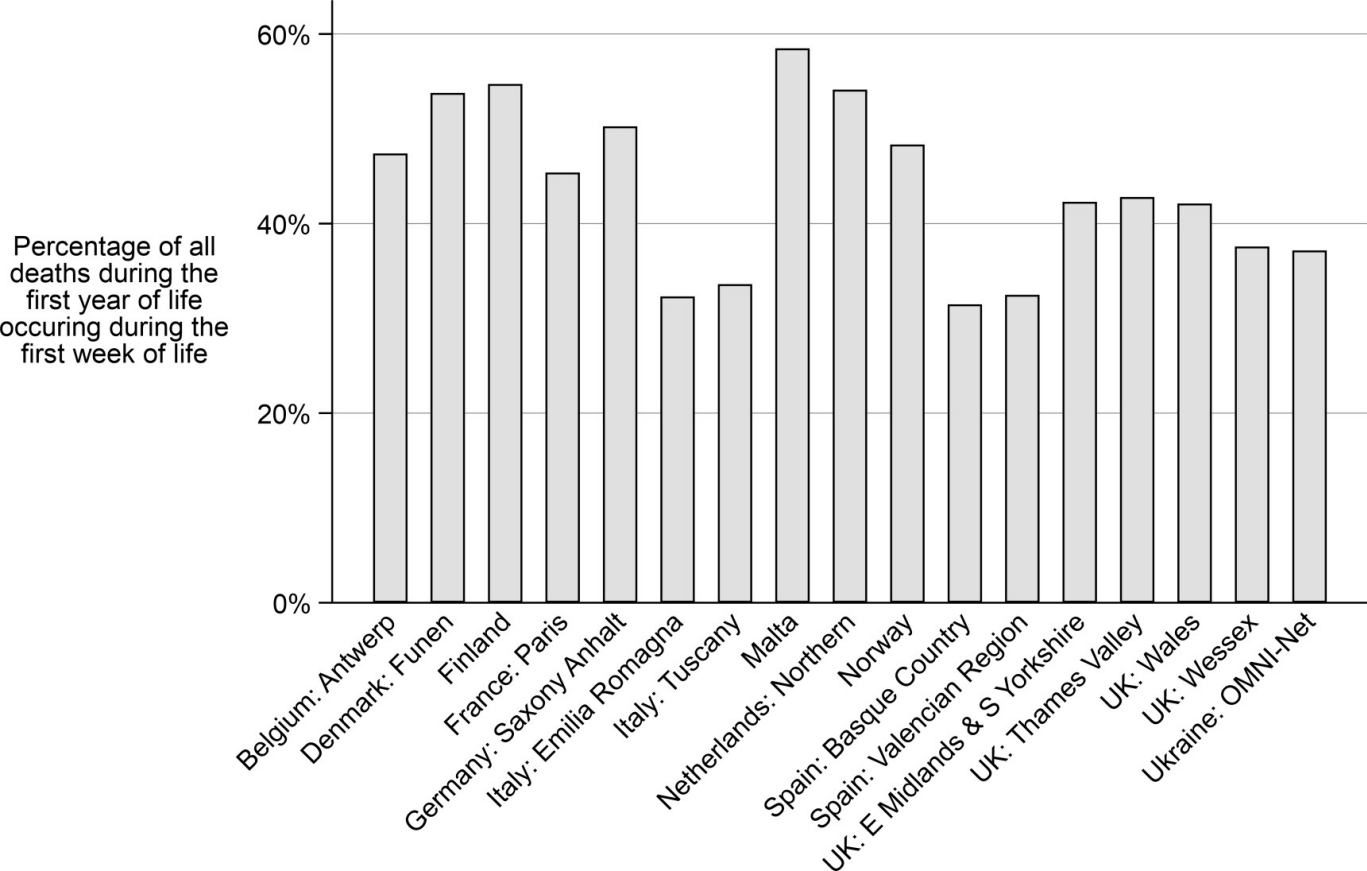
Linked deaths occurring during the first week as a percentage of deaths occurring during the first year of life according to registry.

Table 2 and Fig 1 show the linkage success for registries linking to vital statistics. Two registries (Norway, and Denmark: Funen) were able to link all cases for all years; Finland was able to link over 99.9% of cases but 60 cases had incorrect ID numbers so they could not be linked with vital statistics. Paris linked over 99% of cases for all years, Wales and the Northern Netherlands linked over 95% of their cases. The two Italian registries (Emilia Romagna and Tuscany) and all three UK English registries were unable to link >85% of cases in the earlier years (Fig 1). The proportion of linked deaths during the first week of life out of all deaths in the first year of life were lower in the Italian and Spanish registries which indicates potential data linkage issues (Fig 2).

**Table 2:** Linkage and follow up performance for registries linking their data to national vital statistics.

Country: Registry	Earliest years of birth	Children with CA	Linked births (% all births)	Not linked births (% all births)	Births with incomplete follow up* (% all births)	Deaths in linked births (% linked births)	Known deaths in unlinked births† (% unlinked births)	Notes including reasons not linked
Denmark: Funen	1995	2,425	2,425 (100)	0 (0)	63 (2.6)	149 (6.1)	0 (0)-	
Finland.	1995	42,921	42,861 (99.9)	60 (0.1)	218 (0.5)	1,770 (4.1)	0 (0)	Non-linkage occurred when cases had incorrect or incomplete PINs
France: Paris	1997	11,724	11,623 (99.1)	101 (0.9)	24 (0.2)	585(5.0)	0 (0)	Non-linkage occurred when there was no match on unique ID and child’s date of birth
Italy: Emilia Romagna	1995	8,019	7,327 (91.4)	692 (8.6)	N/A	256 (3.5)	45 (6.5)	Errors in SDO ID numbers, errors in the registration of the Fiscal Code from which the child identification number is created, some children not registered with CeDAP
Italy: Tuscany	1995	5,951	5,187 (87.2)	764 (12.8)	75 (1.4)	147 (2.8)	46 (6.0)	Invalid ID, due to one of the 5 matching variables being incorrect
Netherlands: Northern	1995	8,605	8,325 (96.7)	280 (3.3)	105 (1.2)	551 (6.6)	74 (26.0)	Using date of birth, sex, postal code (6 digits) and year of validity of the postal code, did not result in a unique match with encrypted national identification number (rinnumber). From 1995–2012 the coding was done by hand without a rinnumber, with three different codebooks
Norway	1995	27,201	27,201 (100)	0 (0)	448 (1.6)	1034 (3.8)	0 (0)	NA
UK: Thames Valley	1995	4776	4,191 (87.8)	585 (12.2)	319 (6.7)	317 (6.6)	^a^ (1.0)	Insufficient personal identifiers in original register data, e.g. missing NHS Numbers and names. These were often not available for babies who die soon after birth. Names were not always recorded particularly in earlier years. Postcodes were those at birth and not current postcodes.
UK: East Midlands and South Yorkshire	1998	16,363	14,645 (89.5)	1718 (10.5)	799 (4.9)	1251 (7.6)	114 (6.6)	As above
UK: Wessex	1995	7,839	6,774 (86.4)	1065 (13.6)	281 (3.6)	538 (6.9)	39 (3.7)	As above
UK: Wales	1998	18,188	18,128 (99.7)	60 (0.3)	1777 (9.8)	796 (4.4)	49 (81.7)	Non-linkage occurred when a valid NHS number was not present or linkage to the Welsh Demographic Service was unsuccessful

*Incomplete follow up: Children who were lost to follow up/linkage due to adoption or emigration/leaving the region covered by the Vital statistics database.

†Known deaths in unlinked children: Cases known to have died by the EUROCAT registry, but not linked to a mortality record in the vital statistics database.

CA = congenital anomaly, NA = not applicable.

^a^ Number of Known deaths in unlinked births is <8 and hence is suppressed.

The registries were asked to classify the strength of the linkage. The linking institutions for the eleven registries that linked their CA data to vital statistics classified all their matches as strong, with the exception of the UK English registries, where strong matches accounted for between 92% - 99% of all matches.

Table 2 also provides information on the proportion of children who were not followed up for the full 10 years of life or to 31^st^ December 2015 due to adoption or to leaving the region or country covered by the vital statistics database. Ten of the eleven registries that linked to vital statistics had information on loss to follow-up, seven with national coverage (Finland, Norway, Denmark: Funen, UK: Thames Valley, East Midlands, Wessex, and Wales). The Emilia Romagna registry did not have loss to follow-up information. The proportion of births lost to follow-up was under 2% for five registries, 2.6% for Denmark: Funen, 3.6%-6.7% for the UK English registries and 9.8% for Wales.

For four registries (Emilia Romagna, Tuscany, Northern Netherlands, and Wales), the proportion of known deaths occurring in the unlinked cases was higher than the proportion of deaths in the linked cases (Table 2).

### Success of linkage to mortality records

Table 3 shows the numbers of deaths identified by linking the EUROCAT data with mortality records. The success of registries linking to mortality records only cannot be estimated since registry differences in the proportions of deaths amongst all CA cases may be explained by differences in mortality rates in the registries or may reflect the ability to link and the accuracy of the linkage in the registries. Table 3 shows that for three registries (Antwerp, Basque Country, and Valencian Region) around 10% of all deaths were deaths recorded in the EUROCAT registry that had not been linked to the mortality records. In the Valencian Region registry, the majority of the unlinked deaths were premature and were identified in the Perinatal Mortality registry but were not recorded in the mortality registry. Half of the unlinked deaths in the Valencian Region registry died within the first 24–48 hours of life.

**Table 3:** Success of linkage for registries linking their data to mortality records only.

Country: Registry	Earliest years of birth	Children with CA	Total deaths (linked deaths and unlinked known deaths) * (% all live births)	Unlinked known deaths* (% total deaths)	Linked deaths considered “weak” linkage (% all linked deaths)	Notes including reasons not linked
Belgium: Antwerp	1997	7,865	412 (5.2)	55 (11.8)	357 (100)	Only deaths during the first year of life were identified. All linkage considered weak as national id numbers could not be used
Germany: Saxony-Anhalt	1995	8,698	209 (2.4)	0 (0.0)	0 (0.0)	Due to German Statistics Law, the Federal Office of Statistics would not link individual CA case data to their mortality or other records.
Croatia: Zagreb	1995	441	3 (0.9%)	-	-	Analysis of linkage quality was not performed as only 345 of 441 cases (78%) had an identifier, 2011–2014. Years 1995–2010 dropped because no identification numbers.
Malta	1995	2718	238 (8.8)	3 (1.2)	0 (0.0)	Unlinked known deaths not on mortality register due to mortality in first days of life or if death occurred abroad
Spain: Basque Country	1995	5,904	369 (6.2)	42 (10.2)	56 (15.2)	Problems with identification data in the database form 1995–1999 led to very low linking, had to be done manually
Spain: Valencian Region	2007	7,389	366 (5.0)	50 (12.8)	0 (0.0)	The majority of unlinked deaths were premature and were identified in the Perinatal Mortality registry but not in the mortality database; half of the unlinked deaths died within the first 24–48 hours of life
Ukraine: OMNI-Net	2005	5,835	755 (12.9)	0 (0.0)	0 (0.0)	All non-matching IDs were manually reviewed and matched

*Unlinked known deaths: Cases known to have died by the EUROCAT registry, but not linked to a mortality record.

### Potential bias from missed linkages

In registries that linked to vital statistics, characteristics of the live births recorded in the CA registries can be compared to live births that were linked and those that were not to determine if linkage success is associated with any specific risk factors. For registries that linked to mortality records no such comparison is possible. However, EUROCAT registries report survival for the first week of life and many also have survival in the first year of life. Therefore, the characteristics of live births known to have resulted in a death but not linked can be compared to those live births who were linked to the mortality records. This will give an indication of any factors associated with linkage success, but the estimates will be much more imprecise as the sample sizes are much smaller and there is bias as the EUROCAT registries are more likely to have a death recorded if it occurs within the first week of life.

Table 4 shows that when linking to vital statistics, live births were more likely not to be linked if they died within the first week of birth (odds ratio = 3.44; 95% CI: 2.92–4.04). In addition, babies born before 37 weeks and babies with birth weights <2,500 g were more likely not to be linked with odds ratios of around 1.3. Babies to younger mothers and also twins were less likely to be linked. Infant sex was not associated with linkage success. The results from linking to mortality records were very similar, though only statistically significant for deaths within the first week of life (odds ratio 3.44; 95%CI 2.23–5.30). Fig 2 plots, the linked deaths occurring during the first week of life as a percentage of all deaths occurring during the first year of life. Those registries with high linkage rates to vital statistics recorded over 40% of deaths occurring in the first week of life. Registries below 40% included those with poor linkage to vital statistics and those linking only to mortality records.

**Table 4:** Comparison of linkage failure according to characteristics of the mother and baby (i) in all births in nine registries linking to vital statistics† and (ii) in all births resulting in a death in four registries linking to mortality records‡.

Variable	Category	Odds (95% CI) of live births not being linked compared to baseline†	Odds (95% CI) of deaths not being linked compared to baseline‡
Maternal age (years)	<20	1.73(1.54–1.94)	4.17 (1.47–11.85)
	20–34	1	1
	≥35	0.82(0.76–0.89)	0.90 (0.56–1.45)
Gestational age at delivery (weeks)	24–27	1.2(0.88–1.63)	2.07 (0.90–4.8)
	28–31	1.55(1.31–1.83)	1.67 (0.85–3.28)
	32–36	1.21(1.11–1.32)	1.26 (0.76–2.09)
	≥37	1	1
Number of babies	Singleton	1	1
	Multiple	1.22(1.06–1.42)	0.74 (0.36–1.52)
Infant sex	Male	1	1
	Female	0.99(0.93–1.05)	1.19 (0.78–1.82)
Survival in 1^st^ week	Survived 1^st^ Week	1	1
	Died within 1^st^ week	3.44(2.92–4.04)	3.44 (2.23–5.3)
Birth weight (g)	<1000	1.37(1.06–1.77)	1.29 (0.57–2.96)
	1000–1499	1.37(1.14–1.64)	1.22 (0.57–2.61)
	1500–2499	1.21(1.11–1.32)	1.06 (0.66–1.71)
	2500–3999	1	1
	≥4000	0.95(0.83–1.09)	0.42 (0.05–3.39)

†: Registries included: Finland, Paris, Emilia Romagna, Tuscany, Northern Netherlands, Wales, Thames Valley, Wessex, East Midlands and South Yorkshire; Excluded registries: Norway and Denmark: Funen as no unlinked live births.

‡: Registries included: Basque Country, Valencian Region, Malta, and Antwerp; Excluded registries: Saxony Anhalt and Ukraine as no known unlinked deaths.

### Accuracy of linked variables

**Fig 3:**
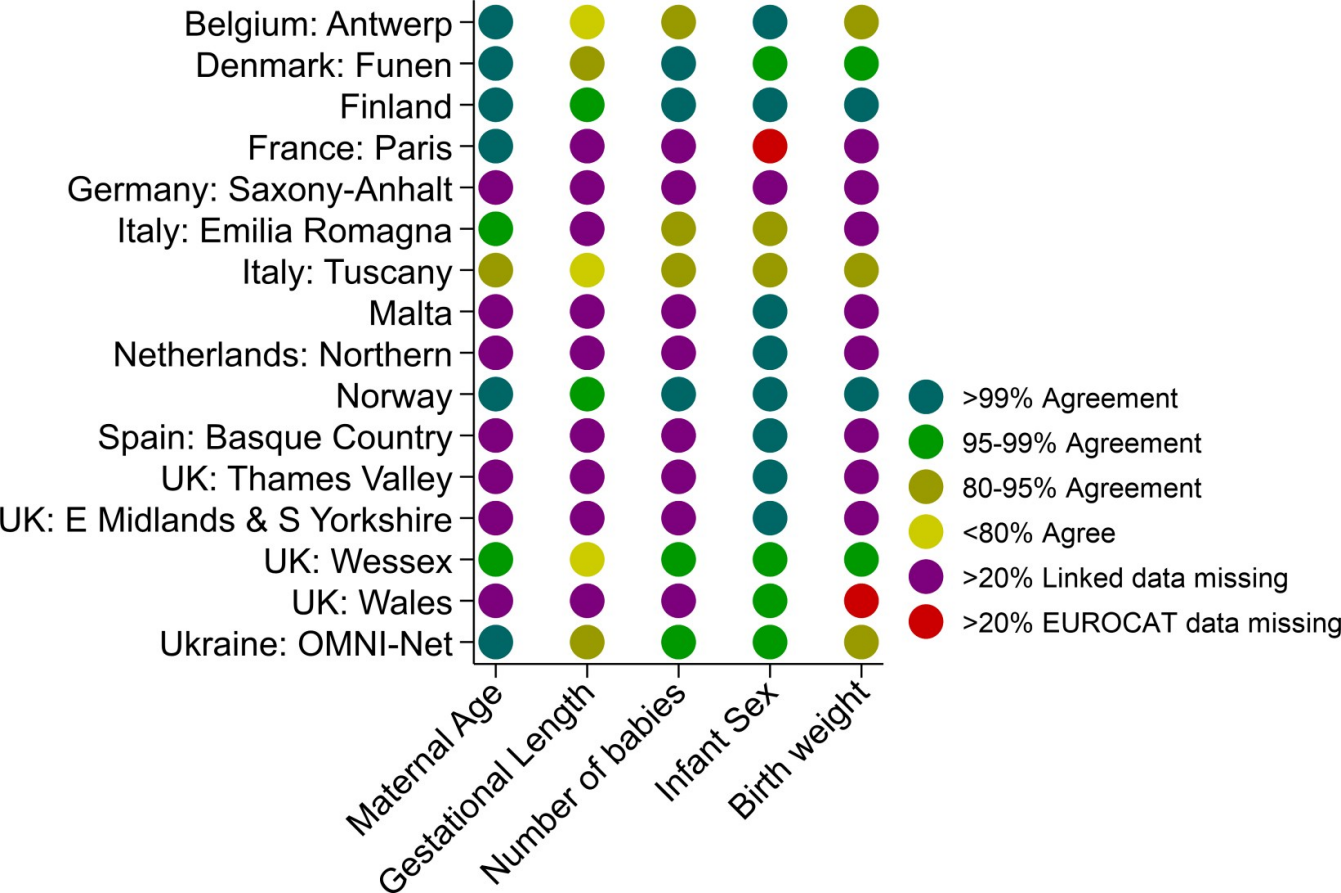
Accuracy of linked variables by registry.

Fig 3 compares the values of specific variables in the EUROCAT data and in the linked data. Some of the variables, such as maternal age and infant sex, would have been used to perform the probabilistic linkage. Registries that linked to mortality records only were much more likely to have a large proportion of data missing in the mortality records for maternal age, gestational length, number of babies and birth weight, as this information is not normally recorded on death certificates unless the region has a separate death certificate for recording neonatal/infant deaths. The agreement was very good for maternal age and infant sex. The EUROCAT variable for infant sex was not included in the Paris CA case file. The accuracy and completeness of most variables improved over time in four registries in whom the overall accuracy and completeness was lower.

## Discussion

We report the accuracy and completeness of record linkage when linking CA registry data to national vital statistics or mortality records in 18 registries in 13 European countries to examine survival of children born with a CA over a 20-year period from 1995 to 2014. For registries linking to vital statistics, the accuracy of the linkage was assessed over time and was shown to be excellent for Finland, Norway, and Denmark: Funen and good for Paris, Wales, and the Northern Netherlands, with very few children having incomplete follow-up periods. Although the linkage improved over time for the two Italian and three UK English registries, they were unable to link at least 85% of all live born cases in the early years. As a result, Italian and English data for the early years will be excluded from future analyses, as it was not sufficiently accurate. In contrast, it was extremely difficult to assess the accuracy of the linkage for registries that only linked to mortality records.

For both types of linkage there was an indication that live births resulting in deaths within the first week of life were less likely to be linked. Preterm births and those with low birthweights were also less likely to be linked, possibly as these are risk factors for neonatal deaths. A low proportion of deaths occurring in the first week of life compared to the first year of life, particularly if below 40%, may be an indication of unsuccessful matching, regardless of the type of linkage. For Saxony-Anhalt, another indication that some deaths may be unlinked was that the survival, particularly of anomalies associated with high fatality rates, was significantly higher than that of any other registry [14].

There are several reasons why early deaths, particularly those occurring during the first hours and days of life, were less likely to be matched. Firstly, assigning national ID numbers can take several days and may not be completed before the death certificates are completed. Secondly, if the child dies within minutes of birth they may also be incorrectly classified as a stillbirth or even a spontaneous abortion (for extremely preterm births with uncertain last menstrual periods) and hence may not receive an ID number. Thirdly, a birth in a maternity unit immediately transferred to a neonatal intensive care unit, possibly in another region, where the child dies may not be linked. Studies have shown that those who die in the first week are less likely to receive a death certificate than those who die later. Also, extremely preterm newborn babies are less likely to get either birth or death certificates compared to full-term newborn babies, even in high-income countries [15,16].

Overall, only five registries distinguished between strong and weak links because for most other registries a successful match required exact agreement on several identifiers, such that all matches were by definition strong. Of these two registries linked to mortality records and three are the UK English registries linking to the same Vital Statistics. Linkages defined as “weak” in one registry were reclassified as “not linked”. One registry classified all their links as weak due to permission not being given to use a unique national ID for matching. The UK English linkage score measures the strength of match to a hospital admissions database but all matched individuals have already been successfully traced through the personal demographics service. In the context of this study, a measure of linkage strength did not appear to be useful.

If a child with a CA was linked, the linked data, if present, were found to be accurate in most registries for maternal age, gestational length (except for Tuscany, Antwerp, and Wales), multiple birth status, infant sex, and birth weight. For governance reasons, Wales is only able to provide week of birth, which explains the lower accuracy found between the Welsh EUROCAT and linked variables for gestational age. In nine registries, more than 20% of information was missing for at least one variable in the linked mortality data. With the exception of infant sex, the other linked data for the UK English registries (extracted from hospital birth records) were missing more than 20% overall. Valencian Region was excluded from this analysis as their mortality records held no information on these variables. In all registries, the accuracy and completeness improved over time.

Studies involving data from the Nordic countries, where unique national ID numbers are used to identify individuals in their national databases, have obtained the high levels of linkage observed in this study. Comparing the linkage results from this EUROlinkCAT study with those from other countries is difficult as many have not reported any information about the accuracy of the linkage [17]. Some studies have made general comments such as “There may have been deaths that could not be tracked due to limitations in administrative data linkages, or if they occurred outside the programme surveillance area” but they did not quantify the proportions of deaths missed [7].

Other studies have examined the survival of children with CAs by linking to mortality records [18]. In a study linking cases in birth defects surveillance programs to death certificate data files in the US, the authors concluded that “There was a potential for incomplete ascertainment of deaths possibly from missed matches of the study cohort to state death certificate files or under ascertainment of out of state deaths”. Again, the authors did not quantify the proportion of deaths that may have been missed.

Future studies planning identification of mortality during and after the neonatal period via linkage with mortality records should take into account that linkage to vital statistics is the method of choice. Linkage to mortality records alone does not enable an accurate assessment of linkage quality to be performed. There was evidence that poor linkage could bias survival estimates as those deaths occurring in the first week of life were less likely to be linked. Therefore, the accuracy and completeness of information must be considered when determining the inclusion of data into an analysis.

## Supporting information

S1 File List of EUROCAT congenital anomaly subgroups used in the survival study. (DOCX)
