# Supplementary material for: Linking a European cohort of children born with congenital anomalies to vital statistics and mortality records: A EUROlinkCAT study
Source: PLoS One. 2021 Aug 27;16(8):e0256535. doi: 10.1371/journal.pone.0256535 (PMC8396745; doi:10.1371/journal.pone.0256535)
Supplement: S1 File — (DOCX) [file pone.0256535.s001.docx]

S1 file. List of EUROCAT congenital anomaly subgroups used in the survival study.

| **EUROCAT Subgroups** | **ICD10-BPA** | **ICD9-BPA†** | **Comments** | **Subgroup binary variable number (al)** |
| --- | --- | --- | --- | --- |
| **All anomalies *** | Q-chapter, D215, D821, D1810^^^, P350, P351, P371 | 74, 75, 27910, 2281^^^, 76076, 76280, 7710, 7711, 77121 |  | al1 |
| **Structural anomalies** |  |  |  |  |
| Spina Bifida | Q05 | 741 | Exclude if associated with anencephalus or encephalocele subgroups | al6 |
| Hydrocephalus | Q03 | 7423 | Exclude hydranencephaly 74232. Exclude association with NTD subgroup | al7 |
| Severe microcephaly | Q02 | 7421 | Exclude association with NTD subgroup | al8 |
| Congenital cataract | Q120 | 74332 |  | al13 |
| Congenital Heart Defects | Q20-Q26 | 745, 746, 7470-7474 | Exclude PDA with GA <37 weeks  Exclude peripheral pulmonary artery stenosis with GA < 37 weeks | al17 |
| Severe CHD | Q200, Q201, Q203, Q204, Q212, Q213, Q220, Q224, Q225, Q226, Q230, Q232, Q233, Q234, Q251, Q252, Q262 | 74500, 74510, 7452, 7453, 7456, 7461, 7462, 74600, 7463, 7465, 7466, 7467, 7471, 74720, 74742 | ICD9-BPA has no code for HRH and double outlet right ventricle | al97 |
| Transposition of great vessels | Q203 | 74510 |  | al19 |
| VSD | Q210 | 7454 |  | al21 |
| ASD | Q211 | 7455 |  | al22 |
| AVSD | Q212 | 7456 |  | al23 |
| Tetralogy of Fallot | Q213 | 7452 |  | al24 |
| Pulmonary valve stenosis | Q221 | 74601 |  | al27 |
| Aortic valve atresia/stenosis | Q230 | 7463 | ICD9-BPA has no code for atresia | al29 |
| Mitral valve anomalies | Q232, Q233 | 7465, 7466 |  | al110 |
| Hypoplastic left heart | Q234 | 7467 |  | al30 |
| Coarctation of aorta | Q251 | 7471 |  | al32 |
| PDA as only CHD in term infants (GA +37 weeks) | Q250 | 7470 | Livebirths only | al100 |
| Cystic adenomatous malf of lung | Q3380 | No code |  | al36 |
| Cleft lip with or without cleft  palate | Q36, Q37 | 7491, 7492 |  | al102 |
| Cleft palate | Q35 | 7490 |  | al103 |
| Oesophageal atresia with/ without trachea-oesophageal fistula | Q390-Q391 | 75030-75031 |  | al41 |
| Duodenal atresia or stenosis | Q410 | 75110 |  | al42 |
| Atresia or stenosis of other parts of small intestine | Q411-Q418 | 75111-75112 |  | al43 |
| Ano-rectal atresia and stenosis | Q420-Q423 | 75121-75124 |  | al44 |
| Diaphragmatic hernia | Q790 | 75661 |  | al48 |
| Gastroschisis | Q793 | 75671 |  | al50 |
| Omphalocele | Q792 | 75670 |  | al51 |
| Multicystic renal dysplasia | Q6140, Q6141 | 75316 |  | al54 |
| Cong hydronephrosis | Q620 | 75320 |  | al55 |
| Hypospadias | Q54 | 75260 |  | al59 |
| Limb reduction defects | Q71-Q73 | 7552-7554 |  | al62 |
| Craniosynostosis | Q750 | 75600 |  | al75 |
| **Chromosomal anomalies** |  |  |  |  |
| Down syndrome | Q90 | 7580 | With or without al17 and al40 | Al89 |
| **All subgroups below analysed as rare** |  |  |  |  |
| **Chromosomal anomalies** |  |  |  |  |
| Trisomy 13 | Q914-Q917 | 7581 |  | Al90 |
| Trisomy 18 | Q910-Q913 | 7582 |  | Al91 |
| Turner syndrome | Q96 | 75860, 75861,  75862, 75869 |  | Al92 |
| Klinefelter syndrome | Q980-Q984 | 7587 |  | Al93 |
| **Rare structural anomalies with a EUROCAT subgroup** |  |  |  |  |
| Encephalocele | Q01 | 7420 | Exclude if ass with anencephalus subgroup | al5 |
| Arhinencephaly / holoprosencephaly | Q041, Q042 | 74226 |  | al9 |
| Anophthalmos / microphthalmos | Q110, Q111, Q112 | 7430, 7431 |  | al11 |
| Anophthalmos | Q110, Q111 | 7430 |  | al12 |
| Congenital glaucoma | Q150 | 74320 |  | al14 |
| Anotia | Q160 | 74401 |  | al16 |
| Common arterial truncus | Q200 | 74500 |  | al18 |
| Double outlet right ventricle | Q201 | No code |  | al109 |
| Single ventricle | Q204 | 7453 |  | al20 |
| Triscuspid atresia and stenosis | Q224 | 7461 |  | al25 |
| Ebstein’s anomaly | Q225 | 7462 |  | al26 |
| Pulmonary valve atresia | Q220 | 74600 |  | al28 |
| Hypoplastic right heart | Q226 | No code |  | al31 |
| Aortic atresia / interrupte aortic arch | Q252 | 74720 |  | al111 |
| Total anom pulm venous return | Q262 | 74742 |  | al33 |
| Choanal atresia | Q300 | 7480 |  | al35 |
| Hirschsprung’s disease | Q431 | 75130-75133 |  | al45 |
| Atresia of bile ducts | Q442 | 75165 |  | al46 |
| Annular pancreas | Q451 | 75172 |  | al47 |
| Indeterminate sex | Q56 | 7527 |  | al60 |
| Situs inversus | Q893 | 7593 |  | al79 |
| VATER/VACTERL | Q8726 | 759895 |  | al112 |
| **New subgroups for EUROlinkCAT** |  |  |  |  |
| **Structural anomalies** |  |  |  |  |
| Anomalies of corpus callosum | Q040 | 74221 |  | aud1 |
| Anomalies of intestinal fixation | Q433 | 7514 |  | aud3 |
| Unilateral renal agenesis | Q600 | No code |  | aud4 |
| Accessory kidney | Q630 | 75330 |  | aud5 |
| Bladder exstrophy | Q641 | 7535 |  | aud6 |
| Epispadia | Q640 | 75261 |  | aud7 |
| Posterior urethral valves | Q6420 | 75360 |  | aud8 |
| Prune Belly | Q794 | 75672 |  | aud9 |
| Arthrogryposis multiplex congenita | Q743 | 75580 |  | aud10 |
| **Genetic syndromes** |  |  |  |  |
| Di George syndrome | D821 | 27910 |  | aud14 |
| Goldenhar syndrome | Q8704 | 75606 |  | aud15 |
| Cornelia de Lange syndrome | Q8712 | 759821 |  | aud16 |
| Noonan syndrome | Q8714 | 759896 |  | aud17 |
| Prader-Willi | Q8715 | 759872 |  | aud18 |
| Beckwith Wiedeman syndrome | Q8730 | 759874 |  | aud20 |
| Williams syndrome | Q8784 | No code |  | aud21 |
| Angelman syndrome | Q8785 | No code |  | aud22 |
| **Chromosomal anomalies** |  |  |  |  |
| Wolff-Hirschorn syndrome | Q933 | 75832 |  | aud23 |
| Cri-du chat syndrome | Q934 | 75831 |  | aud24 |
| Karyotype XXX | Q970 | 75885 |  | aud25 |
| **Sequences** |  |  |  |  |
| Pierre-Robin sequence | Q8708 | 75603 |  | aud27 |

*All Anomalies = ALL cases of congenital anomaly, excluding cases with only minor anomalies as defined in Section 3.2 in Guide 1.4 for cases born post-2005. Cases with more than one anomaly are only counted once in the “All Anomalies” subgroup.

†EUROCAT ICD-9 codes are used with the British Paediatric Association (BPA) extension code: <http://www.eurocat-network.eu/content/EUROCAT-ICD9-with-BPA-Extension.pdf>
